# Mitochondrial Dynamics in the *Drosophila* Ovary Regulates Germ Stem Cell Number, Cell Fate, and Female Fertility

**DOI:** 10.3389/fcell.2020.596819

**Published:** 2021-01-28

**Authors:** Marcia Garcez, Joana Branco-Santos, Patricia C. Gracio, Catarina C. F. Homem

**Affiliations:** ^1^iNOVA4Health, CEDOC, NOVA Medical School, NMS, Universidade Nova de Lisboa, Lisbon, Portugal; ^2^Graduate Program in Areas of Basic and Applied Biology (GABBA), Universidade do Porto, Porto, Portugal

**Keywords:** mitochondrial dynamics, germ stem cell, oxidative phosphorylation, differentiation, oogenesis, fertility, *Drosophila melanogaster*

## Abstract

The fate and proliferative capacity of stem cells have been shown to strongly depend on their metabolic state. Mitochondria are the powerhouses of the cell being responsible for energy production *via* oxidative phosphorylation (OxPhos) as well as for several other metabolic pathways. Mitochondrial activity strongly depends on their structural organization, with their size and shape being regulated by mitochondrial fusion and fission, a process known as mitochondrial dynamics. However, the significance of mitochondrial dynamics in the regulation of stem cell metabolism and fate remains elusive. Here, we characterize the role of mitochondria morphology in female germ stem cells (GSCs) and in their more differentiated lineage. Mitochondria are particularly important in the female GSC lineage. Not only do they provide these cells with their energy requirements to generate the oocyte but they are also the only mitochondria pool to be inherited by the offspring. We show that the undifferentiated GSCs predominantly have fissed mitochondria, whereas more differentiated germ cells have more fused mitochondria. By reducing the levels of mitochondrial dynamics regulators, we show that both fused and fissed mitochondria are required for the maintenance of a stable GSC pool. Surprisingly, we found that disrupting mitochondrial dynamics in the germline also strongly affects nurse cells morphology, impairing egg chamber development and female fertility. Interestingly, reducing the levels of key enzymes in the Tricarboxylic Acid Cycle (TCA), known to cause OxPhos reduction, also affects GSC number. This defect in GSC self-renewal capacity indicates that at least basal levels of TCA/OxPhos are required in GSCs. Our findings show that mitochondrial dynamics is essential for female GSC maintenance and female fertility, and that mitochondria fusion and fission events are dynamically regulated during GSC differentiation, possibly to modulate their metabolic profile.

## Introduction

Metabolic plasticity, in particular the balance between glycolysis and oxidative phosphorylation (OxPhos), has been shown to regulate cell fate both in stem cells and in their differentiated lineages, across several models (Tsogtbaatar et al., 2020). High glycolytic flux and low mitochondria content have been observed in stem cells, whereas more specialized cells rely mainly on OxPhos to meet their metabolic demands and have higher numbers of mitochondria (Rafalski et al., 2012). Mitochondria are central organelles in metabolism regulation, with several key metabolic pathways, such as the Tricarboxylic Acid Cycle (TCA), lipid beta oxidation, and OxPhos, occurring in these organelles. Mitochondria are also important regulators of Ca^2+^ homeostasis and apoptosis among other processes (Nunnari and Suomalainen, 2012). Mitochondria function is tightly linked to their morphology that is modulated through events of fusion and fission between their inner and outer membranes, a process known as mitochondrial dynamics. While predominance of fission events is associated with smaller and more punctate mitochondria, shifting the balance toward fusion leads to larger and more aggregated mitochondria (Spurlock et al., 2020). These changes in mitochondria morphology occur rapidly in response to changes in metabolic requirements or external signals (Zhang et al., 2018).

However, it is not clear how stem cell fate and specific metabolic profiles are associated with mitochondria morphology. To address this question, we took advantage of the well-characterized ovarian germ stem cell (GSC) lineage in *Drosophila*. Female GSCs are located in a simple anatomical structure known as germarium and are among a few of the stem cells present in adult tissues. GSCs can be reliably identified, and their lineages well-characterized and easily traced. This, together with the large availability of genetic tools for their manipulation, makes them one of the best models to study stem cell biology.

Mitochondria in female GSCs are of particular importance because, in addition to their role in metabolic regulation, these cells provide the only pool of mitochondria that will be inherited by the progeny, since male mitochondrial DNA (mtDNA) is eliminated during spermatogenesis (DeLuca and O'Farrell, 2012). Mitochondrial dynamics plays an important role in ensuring the quality of mitochondria to be inherited by the progeny. While fusion events enable mixing of matrix components between mitochondria, promoting their homogenization and a healthy mitochondria pool (Chan, 2012), mitochondrial fission was shown to be crucial for selection of mitochondria without deleterious mtDNA (Lieber et al., 2019). Although male mitochondria do not contribute to progeny, mitochondrial dynamics has been shown to be important in the early stages of spermatogenesis, with disruptions in this mechanism causing defects in GSC number or spermatogenesis arrest (Demarco et al., 2019; Varuzhanyan et al., 2019).

Previous studies of *Drosophila* female GSCs showed that mitochondria morphology changes during GSC lineage differentiation (Cox and Spradling, 2003), suggesting that mitochondrial dynamics may play an important role in fate regulation. It has also been shown that female GSCs have reduced mitochondrial membrane potential (Wang et al., 2019) and reduced levels of electron transport chain (ETC) proteins (Kai et al., 2005) when compared with more differentiated germ cells. These results suggest that mitochondria play an unimportant role in GSCs, and that they only become important for the increase in OxPhos that occurs with differentiation. Despite these observations suggesting that the metabolic profile of GSC lineages correlates with their fate and potency, it is still not known if mitochondria play a role in GSCs. Furthermore, it is not clear if there is a functional link between mitochondria morphology and the fate of GSC lineages.

**Figure 1:**
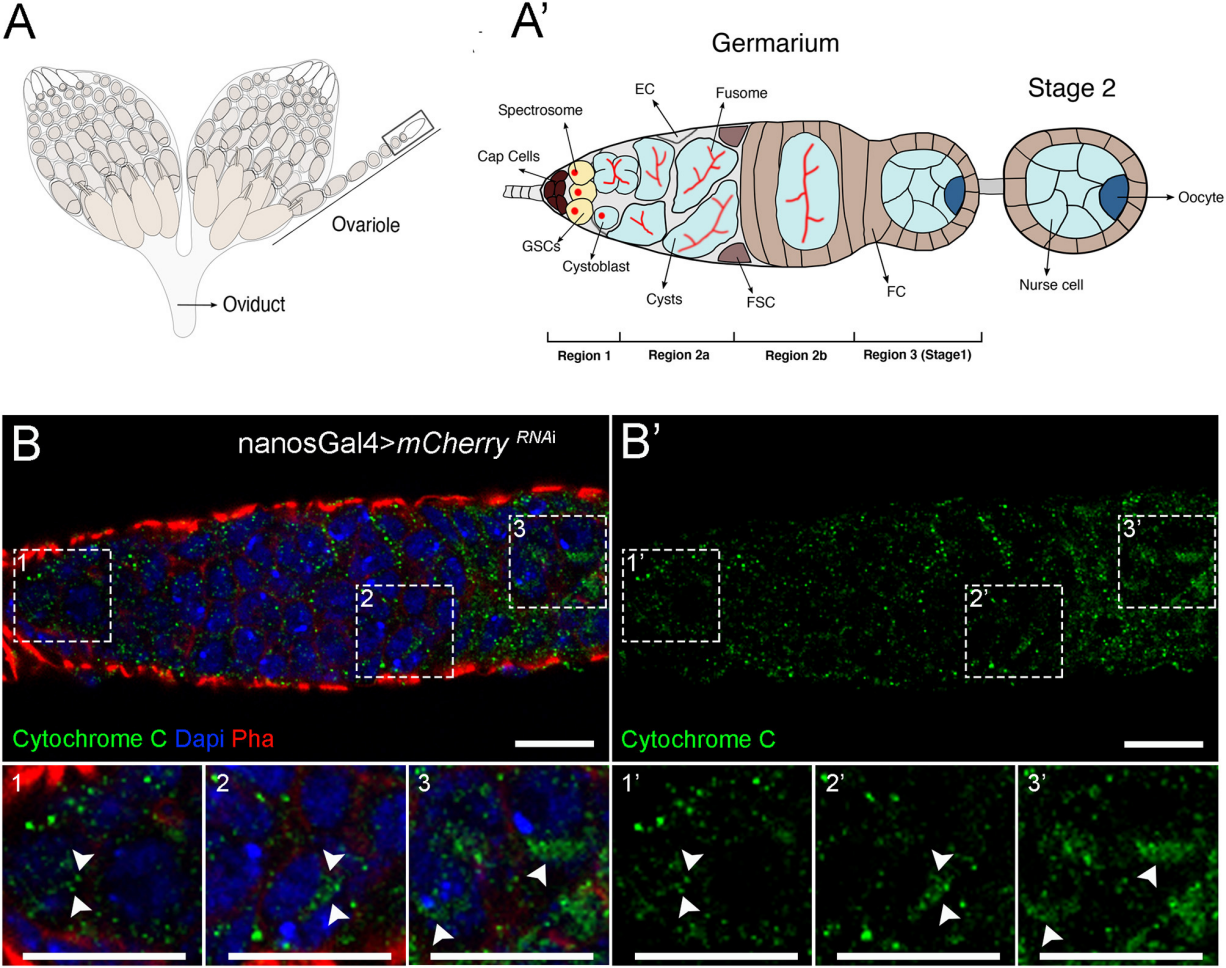
Mitochondria undergo changes in morphology during cellular differentiation in the adult germarium. **(A)** Schematic representation of *Drosophila* ovaries illustrating the different stages of egg formation in the ovarioles. **(A′)** Enlarged view of the germarium. The germarium is located at the most anterior part of the ovariole (black box outlined in **A**). At the most anterior tip of the germarium (region 1) reside the germ stem cells (GSCs). GSCs are connected to niche cells, the cap cells, and are characterized by the presence of round spectrosomes. GSCs divide to self-renew and form one cystoblast that occupies a more posterior position and is not in direct contact with cap cells. The cystoblast starts differentiation and divides to give rise to cysts (region 2a); this process is accompanied by the differentiation of the spectrosome into an elongated structure, the fusome. In region 2b, the cysts are encapsulated by follicle cells (FC), and in region 3, 1 out of the 16 cyst cells is specified as the oocyte (dark blue cell at the posterior side of the cyst), whereas the remaining 15 cells become nurse cells, supportive cells that will aid in oocyte development into one mature egg. Escort cells (ECs) and follicle stem cells (FSCs) are also represented. **(B)** Representative images of a control germarium (expressing *mCherry*^*RNAi*^ driven by *nos*Gal4) showing staining for mitochondria in the different developmental regions. Mitochondria labeled by Cytochrome *c* staining are visualized in green **(B′)**. GSCs mostly display punctate and fragmented mitochondria (1 and 1′) that progressively aggregate during cyst differentiation (2–3 and 2′-3′), suggesting more fusion. Fissed and fused mitochondria in regions 1 (1 and 1′), 2b (2 and 2′), and 3 (3 and 3′) of the germarium are identified with arrowheads in zoomed areas (dashed white squares). Nuclei are labeled with DAPI (blue), and F-actin is labeled with Phalloidin (Pha, red). Scale bars represent 10 μm.

To address the role of mitochondrial dynamics in female GSCs, we interfered with mitochondrial fission and fusion mechanisms in the female germline of *Drosophila* to determine how lineages are affected. Each ovariole contains two to three GSCs located at the tip of the germarium, in a protected microenvironment or niche (Figures 1A,A′). GSCs are connected to niche cells, the cap cells, and are characterized by the presence of the spectrosome (Figure 1A′, region 1). GSCs divide to produce one daughter cell through self-renewal and another daughter cell that is no longer directly connected to cap cells and therefore initiates differentiation, the cystoblast. The cystoblast undergoes four subsequent mitotic divisions with incomplete cytokinesis to generate a cyst of 16 interconnected cells. Cyst differentiation is accompanied by the differentiation of the spectrosome into the fusome, a germline-specific organelle of communication. This cyst is then completely encapsulated by follicle cells (Figure 1A′, regions 2a and 2b), and one cell in this 16-cell cyst is specified as the oocyte, whereas the remaining 15 cells become nurse cells (Figure 1A′, region 3). The surrounding nurse cells support oocyte growth that continues developing in sequentially more mature follicles until a fully developed egg is formed at the posterior end of the ovariole (Figure 1A).

Here, we have characterized mitochondria morphology in GSCs and in their early differentiated lineage in the *Drosophila* germarium. We have also knocked down the regulators of fusion, Mitochondrial Assembly Regulatory Factor (Marf or mitofusin) and Optic Atrophy 1 (Opa1), as well as the regulator of fission Dynamin-related protein 1 (Drp1) in female GSCs, and analyzed how their depletion affects GSC number and lineage. Our results show that the undifferentiated GSCs predominantly have sparse and punctate mitochondria, whereas more differentiated germ cells show more aggregated mitochondria, suggesting more fusion. Dysregulation of mitochondrial dynamics in GSCs, by interfering with fusion or fission mechanisms, leads to a loss of GSCs and causes a severe reduction in female fecundity. In addition, depletion of mitochondrial dynamics regulators in GSCs tends to increase mitochondrial membrane potential that correlates with GSC loss and defects in germline development in a mechanism independent of reactive oxygen species (ROS). Interestingly, reducing the levels of key enzymes in the TCA cycle also leads to a reduction in the number of GSCs, suggesting that mitochondrial dynamics may be required for TCA/OxPhos and, thus, GSC maintenance. Surprisingly, we found that impairment of mitochondrial dynamics strongly affects egg chamber structural organization, ultimately causing arrest of egg chamber development at late stages and consequently reduced oogenesis. Overall, our results show that mitochondria, and specifically TCA/OxPhos metabolism, play an essential role in the maintenance of GSCs contradicting the notion that these are mostly required in differentiated cells. Additionally, our work uncovers a novel role for mitochondrial dynamics in the regulation of egg chamber development and female fertility.

## Materials and Methods

### Fly Husbandry and Stocks

Flies were raised and maintained on standard cornmeal medium at 25°C in 12 h light/dark cycle, unless otherwise stated. To identify mitochondrial regulators that impact GSC number, we analyzed UAS-RNAi lines targeting genes mediating mitochondrial fission (*Drp1*) and fusion (*Marf* and *Opa1*) and genes encoding key mitochondrial metabolic enzymes (*Scsalpha1* and *alpha-KGDHC*). The RNAi stocks were obtained from the Bloomington *Drosophila* Stock Center (BDSC) or the Vienna *Drosophila* Resource Center (VDRC) and included the following strains: UAS-*Drp1*^*RNAi*^ (strains BL51483, BL67160, v44156), UAS-*Marf*^*RNAi*^ (strains BL55189, BL67158, v40478, v105261), UAS-*Opa1*^*RNAi*^ (strains BL32358, BL67159, v106290), UAS-*Scsalpha1*^*RNAi*^ (CG1065, v107164), and UAS-*CG5214*^*RNAi*^, referred in text as UAS-*alpha-KGDHC*^*RNAi*^ (v108403). The *nanos*Gal4 line (BL25751) was used to drive the expression of UAS-RNAi transgenes specifically in germ cells. In all experiments, UAS-*mCherry* ^*RNAi*^ (BL35758) was used as a control. Genotypes and sources are detailed in Supplementary Table 1.

### Dissection of Adult *Drosophila* Ovaries

For RNAi-mediated knockdown experiments, crosses of *nanos*Gal4 females with males from RNAi lines were set up. Newly eclosed F1 females were kept in yeast-enriched food for 16 h at 25°C to allow proper ovary maturation. Ovaries were dissected in Schneider's *Drosophila* medium (Gibco) at room temperature as previously described (Gates et al., 2009). Briefly, 5–10 adult flies per genotype were anesthetized with CO_2_, and ovaries were isolated with the aid of forceps. Ovarioles were partially individualized before fixation to facilitate permeabilization.

### Immunostaining and Confocal Microscopy

Ovaries were fixed in 4% paraformaldehyde for 20 min at room temperature and washed 3× with PBST (0.1% Triton X-100 in 1× PBS). Ovaries were blocked using 1% normal goat serum (Jackson Immunoresearch) in 0.1% PBST for at least 20 min at room temperature and incubated overnight at 4°C with primary antibodies (listed below) diluted in blocking solution. Afterwards, ovaries were washed 3×, blocked for 20 min, and incubated for 2 h at room temperature with secondary antibodies (listed below), Alexa Fluor 568 Phalloidin (1:500; Invitrogen) and DAPI (1:1,000; Sigma-Aldrich). Ovaries were then manipulated in 1× PBS using micro dissecting needles (Fine Science Tools) for ovariole individualization and mounted in Aqua-Poly/Mount (Polysciences, Inc.). Images were acquired using a Zeiss LSM880 confocal microscope (Zeiss).

The following primary antibodies were used: mouse monoclonal anti-Hts 1B1 [1:5; Developmental Studies Hybridoma Bank, University of Iowa (DSHB)], rat monoclonal anti-Vasa (1:50; DSHB), and rabbit anti-Cytochrome *c* (1:100; Cell Signaling Technology). The following secondary antibodies (1:1,000; Invitrogen) were used: Alexa Fluor 647-conjugated goat anti-mouse, Alexa Fluor 488-conjugated goat anti-rat, and Alexa Fluor 488-conjugated goat anti-rabbit.

### TMRM and MitoTracker Analysis

To investigate mitochondrial activity, we used a combination of two fluorescent dyes: MitoTracker Deep Red (Life Sciences), to determine the localization of mitochondria, and TMRM [tetramethylrhodamine methyl ester, perchlorate (Biotium)], an indicator of mitochondrial membrane potential. Ovaries were dissected as described above and incubated for 30 min with MitoTracker (500 nM) in Schneider's Drosophila medium at room temperature. Half-way through MitoTracker incubation, TMRM (100 nM) dye was added (15 min incubation period). Ovaries were washed 3× and mounted in PBS. Images of live germaria were acquired immediately after mounting using a Zeiss LSM880 confocal microscope (Zeiss).

### ROS Analysis

Dihydroethidium (DHE) staining was used to probe the levels of ROS. Ovaries were dissected as described above and incubated for 10 min with DHE (30 μM; Life Technologies) and DAPI (1 μg/ml; Sigma-Aldrich) in Schneider's Drosophila medium at room temperature. This was followed by a quick fixation for 5 min in 4% paraformaldehyde after which ovaries were washed once in PBS and mounted in Aqua-Poly/Mount. Germaria were immediately imaged in a Zeiss LSM880 confocal microscope (Zeiss).

### Quantification and Statistical Analysis

All images were analyzed and prepared for publication using the Fiji software (Schindelin et al., 2012).

#### GSC Quantification

Cells were considered GSCs only when the following characteristics were observed: (1) presence of a rounded spectrosome (labeled by anti-Hts antibody) and (2) physical contact with their niche cells in region 1 of the germarium. The average number of GSCs and the corresponding standard deviations (SDs) were calculated for at least 16 germaria per genotype.

Statistical analysis was performed using Prism 6 (GraphPad Software Inc., La Jolla, CA, USA). All data are represented as mean ± SD of at least three independent experiments. Statistical significance of difference to control was calculated using Student's *t*-test and considered significant when *P* < 0.05. Statistically significant differences are depicted as follows: ^*^P < 0.05, ^**^P < 0.01, ^***^P < 0.001, and ^****^P < 0.0001. # indicates *P* < 0.10 and represents a relevant trend of decrease in GSC number. Non-significant (n.s.) differences are *P* > 0.10.

#### TMRM/MitoTracker Quantification

To quantify the average ratio between TMRM and MitoTracker, region 1 of the germarium was delineated using the freehand tool in Fiji, and the mean intensities of TMRM and MitoTracker were measured. TMRM/MitoTracker ratios were calculated by dividing the mean intensity value of TMRM by the one of MitoTracker. Ratiometric images were generated by dividing the TMRM channel by the one of MitoTracker using the Image Calculator in Fiji. The resulting image was pseudo-colored using Rainbow RGB, and a calibration bar was included to facilitate image interpretation. Ratiometric images representative of each genotype are presented. These are in line with the average values of TMRM/MitoTracker ratios calculated for region 1. Statistical analysis was done using Student's *t*-test. Statistically significant differences are depicted as follows: ^*^P < 0.05. # indicates *P* < 0.10 and represents a relevant trend. Data are represented as mean ± SD.

#### Quantification of DHE Labeling

ROS assessment was based on the quantification of nuclear DHE, since when oxidized DHE converts to ethidium, which intercalates within DNA and is therefore within the nucleus (Carter et al., 1994). For cells in region 1 of the germarium with observable nuclear DHE inclusion, the areas of nuclear DHE were delineated using the freehand tool in Fiji, and the mean intensity of fluorescence was measured. Similarly, three sample measurements of the cytoplasmic region surrounding the nucleus were taken and averaged to obtain the cytoplasmic level of DHE. The mean intensity of nuclear DHE level was normalized by the cytoplasmic intensity to account for staining variability. Statistical significance of differences *vs*. control was calculated using *Dunn*'s multiple comparisons. Non-significant (n.s.) differences are when *P* > 0.10. Data are represented as mean ± SD.

### Fecundity Assays

To evaluate egg production, w^1118^ males were crossed to female virgins expressing UAS-*Drp1*^*RNAi*^ (v44156), UAS-*Opa1*^*RNAi*^ (v106290), UAS-*Marf*^*RNAi*^ (v40478), UAS-*Scsalpha1*^*RNAi*^ (CG1065, v107164), and UAS-*CG5214*^*RNAi*^ (referred in text as UAS-*alpha-KGDHC*^*RNAi*^, v108403) or UAS-*mCherry*^*RNAi*^ (BL35758) under the control of *nanos*Gal4. Flies were allowed to courtship and mate for 36 h at 25°C prior to egg counting. Three independent crosses were set up per condition, and flies were kept on fresh laying pots with apple juice agar plates enriched with yeast paste to stimulate egg laying during 3 consecutive days. Agar plates were replaced 3× per day at 3 h interval (plates from overnight periods were discarded). At the end of the day, eggs and female flies were counted. Fecundity was calculated as the number of laid eggs per female per hour.

### qPCR Analysis

Brains from L3 wandering larvae expressing UAS-*Scsalpha1*^*RNAi*^ (v107164) or UAS-*mCherry*^*RNAi*^ (BL35758) driven by actin-GAL4 were dissected in Schneider's Drosophila medium. mRNA was isolated using TRIzol™ LS Reagent (Invitrogen) and treated with TURBO DNA-free™ Kit (Invitrogen™). cDNA was prepared using the RevertAid First Strand cDNA Synthesis Kit (Thermo Scientific™). The following primers were used for amplification:

*Scsalpha1*: GACATGGTGAAGGTGAAGCA and GATGCCGATCTTGCACTGT.
*Act5C*: GATAATGATGATGGTGTGCAGG and AGTGGTGGAAGTTTGGAGTG.

qPCRs were done using GoTaq qPCR Master mix (Promega) on a LightCycler 96 (Roche). Expression of *Scsalpha1* was normalized to *Act5C*, and relative levels were calculated vs. control (*mCherry*^*RNAi*^) using the 2^(−ΔΔCt)^ method (Livak and Schmittgen, 2001). All measurements were done with technical triplicates.

## Results

### Mitochondrial Dynamics Is an Important Regulator of Female GSCs

To clarify the role of mitochondrial dynamics in the female GSC lineage, we started by characterizing mitochondria morphology in GSCs and in their differentiated lineage in the germarium. To analyze mitochondrial morphology, we used an antibody against Cytochrome *c*, a mitochondrial protein present in the intermembrane space of these organelles, commonly used as a mitochondrial marker (Schägger, 2002). Consistently to what had been previously documented by electron microscopy (EM) (Mahowald and Strassheim, 1970; Carpenter, 1975; Cox and Spradling, 2003), we found that GSCs in region 1 have predominantly small punctate mitochondria (Figure 1B, close-up#1), and that mitochondria progressively become more aggregated in regions 2b and 3 consistent with an increase in mitochondria fusion (Figure 1B, close-up#2 and close-up#3, respectively). This increase in mitochondria fusion along cell differentiation suggests that mitochondrial dynamics may play a role in regulating cell fate.

In *Drosophila*, there are two regulators of mitochondria fusion, *Marf* or *mitofusin* that regulates mitochondrial outer-membrane fusion and *Opa1* that mediates fusion of the inner membrane of mitochondria (Pernas and Scorrano, 2016). Mitochondrial fission is mediated by *Drp1* that is recruited to the mitochondrial outer membrane and constricts mitochondria until organelle division occurs (Pernas and Scorrano, 2016). In order to test the hypothesis that mitochondrial dynamics is important for cell fate regulation in the germline, we interfered with both mitochondria fusion and fission regulators and asked whether this affects GSCs. We individually knocked down *Drp1, Marf* , or *Opa1* in germ cells by expressing UAS-RNAi transgenes under the control of *nanos*Gal4 (*nos*Gal4). The UAS/Gal4 system is a method for directing the expression of a genetic element of interest to a specific tissue (Brand and Perrimon, 1993). The Gal4 protein, derived from yeast, serves as a transcriptional activator that binds and activates the upstream activating sequence (UAS), driving the expression of the genetic element under the control of UAS. To induce knockdown, we expressed UAS-dsRNA targeting the genes of interest and simultaneously expressed Gal4 under the control of the *nanos* promotor, which is specifically expressed in the germline.

**Figure 2:**
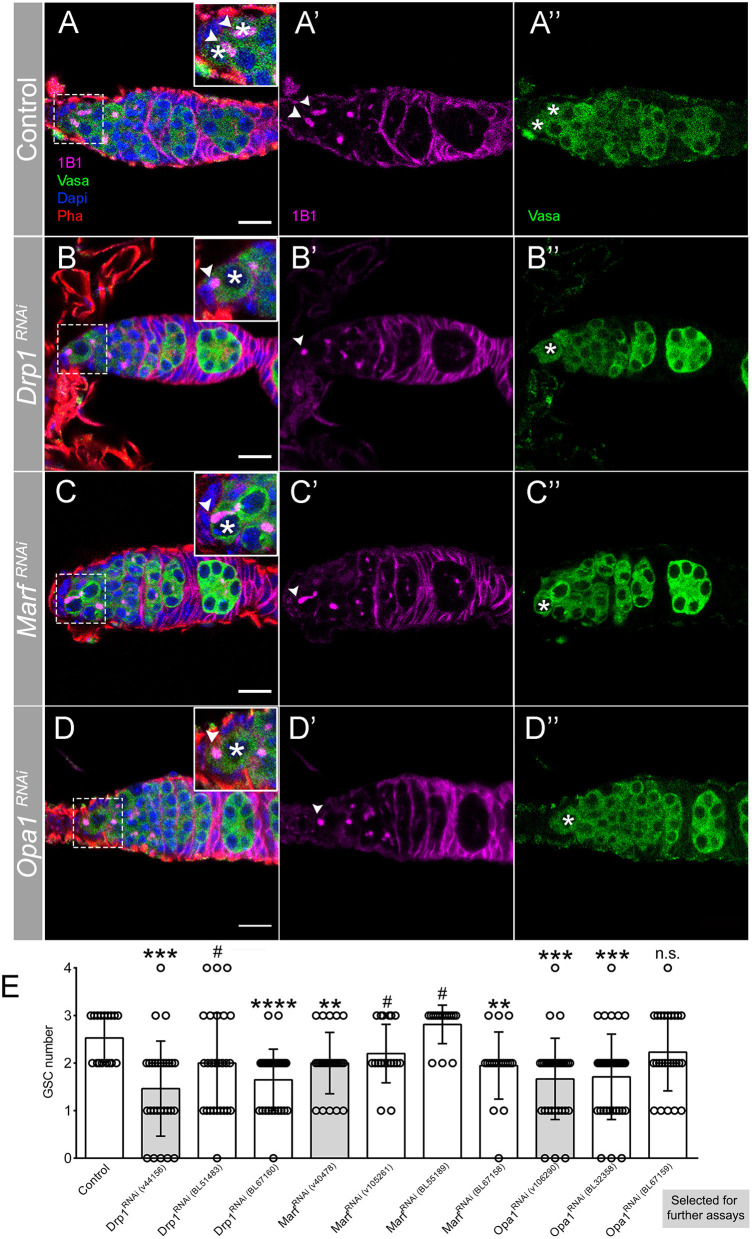
Regulators of mitochondrial fission and fusion are essential for GSC maintenance. **(A–D)** Representative images of adult *Drosophila* germaria expressing RNAi transgenes against *mCherry* (control), *Drp1, Marf* , and *Opa1* by *nos*Gal4. Germ cells are labeled with Vasa antibody (green), and GSCs are marked by the presence of Hts-labeled spectrosome (1B1, pink). The dashed white squares highlight GSCs, marked by asterisks. Arrowheads point to spectrosome of GSCs that are in contact with niche cells. Control germaria typically have 2–3 GSCs **(A–A″,E)**. Depletion of mitochondrial fission regulator *Drp1* **(B–B″,E)** or mitochondrial fusion regulators *Marf* **(C–C″,E)** and *Opa1* **(D–D″,E)** significantly decreases GSC number. Images shown are representative of Drp1^*RNAi*^ (v44156), Marf^*RNAi*^ (v40478), and Opa1^*RNAi*^ (v106290) germaria. Nuclei and F-actin are labeled with DAPI (blue) and Phalloidin (Pha, red), respectively. Scale bars represent 10 μm. **(E)** Quantification of the average number of GSCs (±SD) per germarium expressing the indicated RNAi lines in GSCs (*nos*Gal4). Gray bars indicate the RNAi lines used in the following analyses for each of the mitochondrial dynamics regulators. Statistical significance *vs*. control was calculated using simple sample *t*-test. **P < 0.01; ***P < 0.001; ****P < 0.0001; ^#^*P* < 0.10; n.s., non-significant.

Because there are several RNAi lines available to target each of the mitochondrial dynamics regulatory genes, with variable efficiencies reported (Rai et al., 2014; Sandoval et al., 2014; Deng et al., 2016; Wang et al., 2016; Demarco and Jones, 2019; Demarco et al., 2019; Amartuvshin et al., 2020), we initially analyzed all lines for a possible phenotype in GSCs. While control germaria consistently have 2 or 3 GSCs (Figures 2A,E), 2 out of the 3 RNAi lines used for *Drp1* abrogation show a significant reduction in GSCs, with some germaria having no GSCs present (Figures 2B,E). Knockdown of the outer-membrane fusion regulator *Marf* also leads to a decrease in GSCs number, with 2 out of 4 RNAi lines showing a significant reduction and 2 lines showing a relevant trend toward a decrease (Figures 2C,E). Consistently, downregulation of the inner-membrane fusion regulator *Opa1* significantly reduces GSCs number in 2 out of 3 RNAi lines tested, with some germaria having no GSCs present (Figures 2D,E). Thus, knockdown of mitochondrial dynamics regulators with independent RNAi lines consistently leads to a reduction in GSC numbers, validating the observed phenotypes (see Supplementary Table 2 for detailed characterization). For further analysis of each of these genes, we selected the RNAi line that shows the strongest phenotype and that had been previously validated: *Marf*^*RNAi*^ v40478 (Trevisan et al., 2018; ~80% knockdown), *Opa1*^*RNAi*^ v106290 (Rai et al., 2014; ~70% knockdown), and *Drp1*^*RNAi*^ v44156 (Trevisan et al., 2018; ~70% knockdown, same dsRNA).

**Figure 3:**
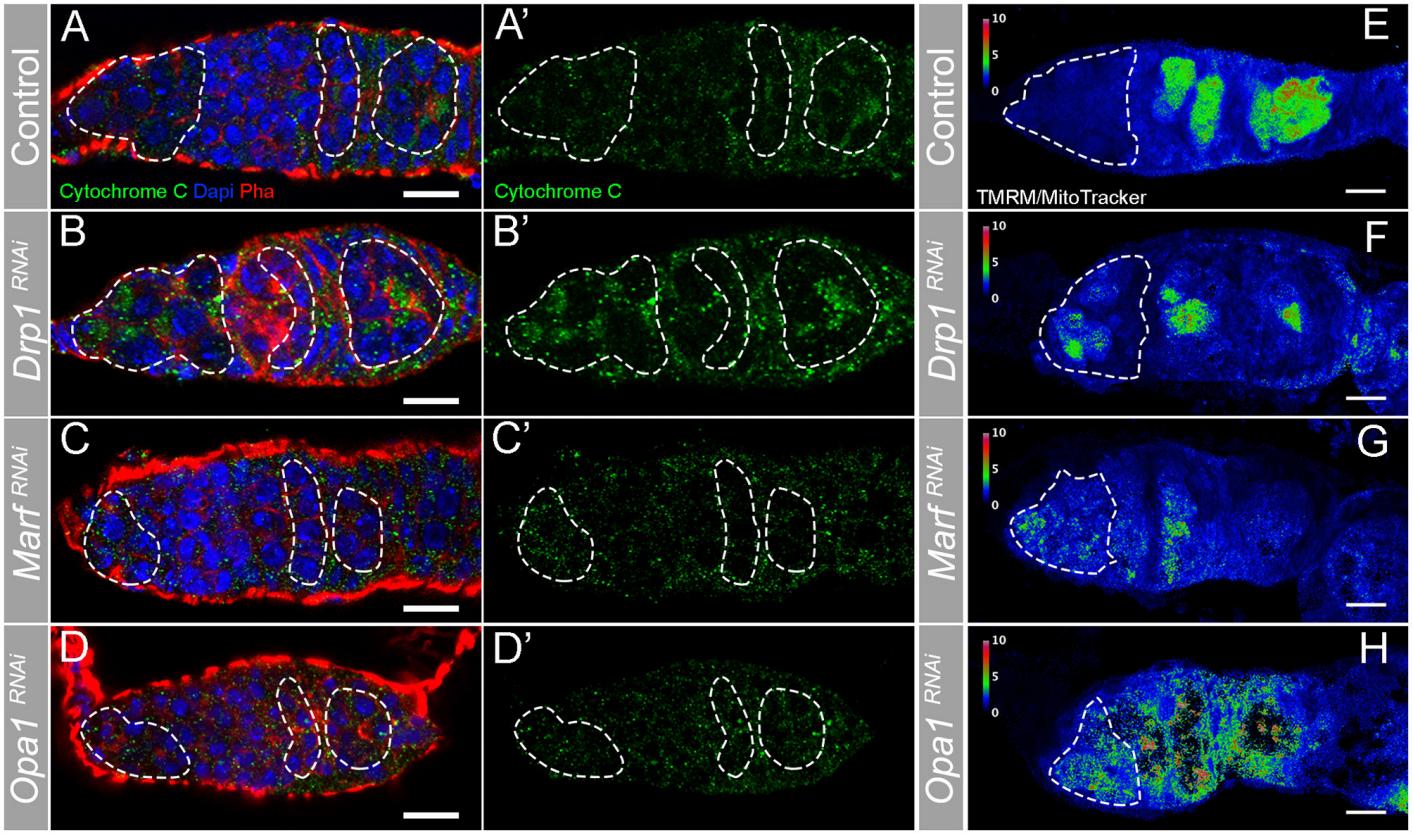
RNAi of *Drp1, Marf* , or *Opa1* disrupts mitochondria morphology in the germarium increasing mitochondrial membrane potential in GSCs. **(A–H)** Representative images of germaria expressing *mCherry*^*RNAi*^, *Drp1*^*RNAi*^, *Marf*^*RNAi*^, and *Opa1*^*RNAi*^ driven by *nos*Gal4. **(A–D)** Mitochondria are labeled by Cytochrome *c* antibody (green). Outlines are indicative of the areas where GSCs (in region 1) and their lineage (in regions 2b and 3) are found to facilitate visual comparison. **(A,A′)** In control germaria, fissed mitochondria are visible in GSCs (region 1), whereas differentiating cysts (regions 2b−3) have progressively more aggregated mitochondria suggesting a higher fusion-to-fission ratio. **(B,B′)** *Drp1*^*RNAi*^ causes hyperfusion of mitochondria in both GSCs (region 1) and differentiating cysts (regions 2b−3). **(C,C′,D,D′)** RNAi of *Marf* or *Opa1* in germ cells leads to a decrease of fused/aggregated mitochondria in regions 2b−3. Nuclei and F-actin are labeled with DAPI (blue) and Phalloidin (Pha, red), respectively. **(E–H)** Ratiometric images of germaria stained with TMRM (indicator of mitochondrial membrane potential) and MitoTracker (indicator of mitochondrial mass). The ratio of TMRM to MitoTracker intensity was calculated in Fiji, and a pseudo-colored image was generated using the rainbow RGB gradient. Outlines indicate region 1, where GSCs are localized. **(E)** Control GSCs have small TMRM/MitoTracker values, indicating low mitochondrial membrane potential. GSCs expressing *Drp1*^*RNAi*^ **(F)**, *Marf*^*RNAi*^ **(G)**, or *Opa1*^*RNAi*^ **(H)** have higher TMRM/MitoTracker ratios than control, indicating that depletion of fission or fusion increases mitochondrial membrane potential. Scale bars represent 10 μm.

To confirm that depleting regulators of mitochondrial dynamics through RNAi successfully modulates mitochondria morphology, we characterized mitochondria morphology in *Drp1*^*RNAi*^, *Marf*^*RNAi*^, and *Opa1*^*RNAi*^ germaria (Figure 3). Compared with control (Figures 3A,A′), germaria expressing *Drp1*^*RNAi*^ show more mitochondria aggregates in region 1, where GSCs are located, as well as in regions 2b and 3 (Figures 3B,B′). This increase in fused-like mitochondria is consistent with a decrease in mitochondrial fission events caused by depletion of fission regulator *Drp1*. In contrast, RNAi of either *Marf* (Figures 3C,C′) or *Opa1* (Figures 3D,D′) causes a reduction of mitochondria aggregation in regions 2b and 3. The decrease in mitochondria aggregation is again consistent with a reduction in fusion events upon depletion of fusion regulators. These results show that interfering with *Drp1, Marf* , and *Opa1* efficiently disrupts mitochondrial dynamics and morphology.

The results showing that interfering with either mitochondria fusion or fission leads to a decrease in GSC number were surprising. One hypothesis to explain these results is that mitochondrial activity might be equally disrupted in both conditions. To test this idea, we measured the mitochondrial inner membrane potential, generated by the ETC complexes. Mitochondria membrane potential is used for the production of ATP being therefore an indicator of mitochondrial respiration (reviewed in Iannetti et al., 2019). Mitochondrial inner membrane potential was measured as the ratio of TMRM (an established indicator of mitochondrial membrane potential) to MitoTracker Deep Red (a marker of mitochondrial mass) (Zhang et al., 2019). This analysis revealed that in control germaria, mitochondria in region 1 have low levels of mitochondrial membrane potential that then increase along germline differentiation (Figure 3E, region 1 outlined), consistent with what has been previously described (Wang et al., 2019). In contrast, in germaria expressing *Drp1*^*RNAi*^ or *Opa1*^*RNAi*^, although variable, mitochondria present in region 1 show increased membrane potential when compared with control (Figures 3E–H, Supplementary Figure 1A). Despite not causing such a strong effect, *Marf*^*RNAi*^ also trends in the same direction with a fraction of the knocked down germaria presenting higher TMRM/MitoTracker values than the control (Figures 3E,G, Supplementary Figure 1A). Interestingly, the change in mitochondrial membrane potential nicely correlates with the severity of the phenotypes observed regarding GSC number. Germaria expressing either *Drp1*^*RNAi*^ or *Opa1*^*RNAi*^ have, on average, one GSC with several germaria presenting no GSCs, whereas *Marf*^*RNAi*^ expressing germaria have on average two GSCs (Figure 2E). These results suggest that interfering with mitochondrial dynamics regulators in GSCs is not unspecifically disrupting mitochondria viability, and that GSCs have all the required components for the effective usage and regulation of their mitochondria. These experiments also reveal that interfering with fusion or fission can lead to increased mitochondrial membrane potential in GSCs, indicating that there is no straightforward connection between mitochondrial dynamics and activity in this context.

Since mitochondria are important sources of ROS, dysregulation of mitochondrial dynamics could also be affecting GSCs by the generation of detrimental ROS levels. Indeed, it was shown that interfering with mitochondrial fission increases the levels of ROS in the *Drosophila* testis (Demarco and Jones, 2019). To evaluate if ROS levels are increased upon knockdown of *Drp1, Marf* , or *Opa1* in GSCs, we used DHE staining, a commonly used method for ROS detection. This analysis has, however, revealed that depletion of any of these genes does not cause a significant change of ROS levels when compared with control (mCherry^*RNAi*^) (Supplementary Figure 1B).

Taken together, our data show that interfering with mitochondrial dynamics, both fusion and fission, compromises GSC pool maintenance leading to a reduction in GSC number. Interestingly, although we found that, in wild-type conditions, germ cells located in region 1 of the germarium have predominantly fissed mitochondria, disruption of fusion mechanisms results in a loss of GSCs, indicating that fusion events play an unexpectedly important role at this stage. Analysis of mitochondrial membrane potential confirmed that mitochondria in wild-type GSCs have low membrane potential that increases along germline differentiation. Altering mitochondrial dynamics in either way leads to increased membrane potential in a fraction of the analyzed germaria, a phenomenon never observed in control germaria. Interestingly, interfering with mitochondrial dynamics in GSCs does not cause a change in the levels of ROS.

### Defective Mitochondrial Dynamics Compromises Ovariole Development and Leads to Reduced Fecundity

**Figure 4:**
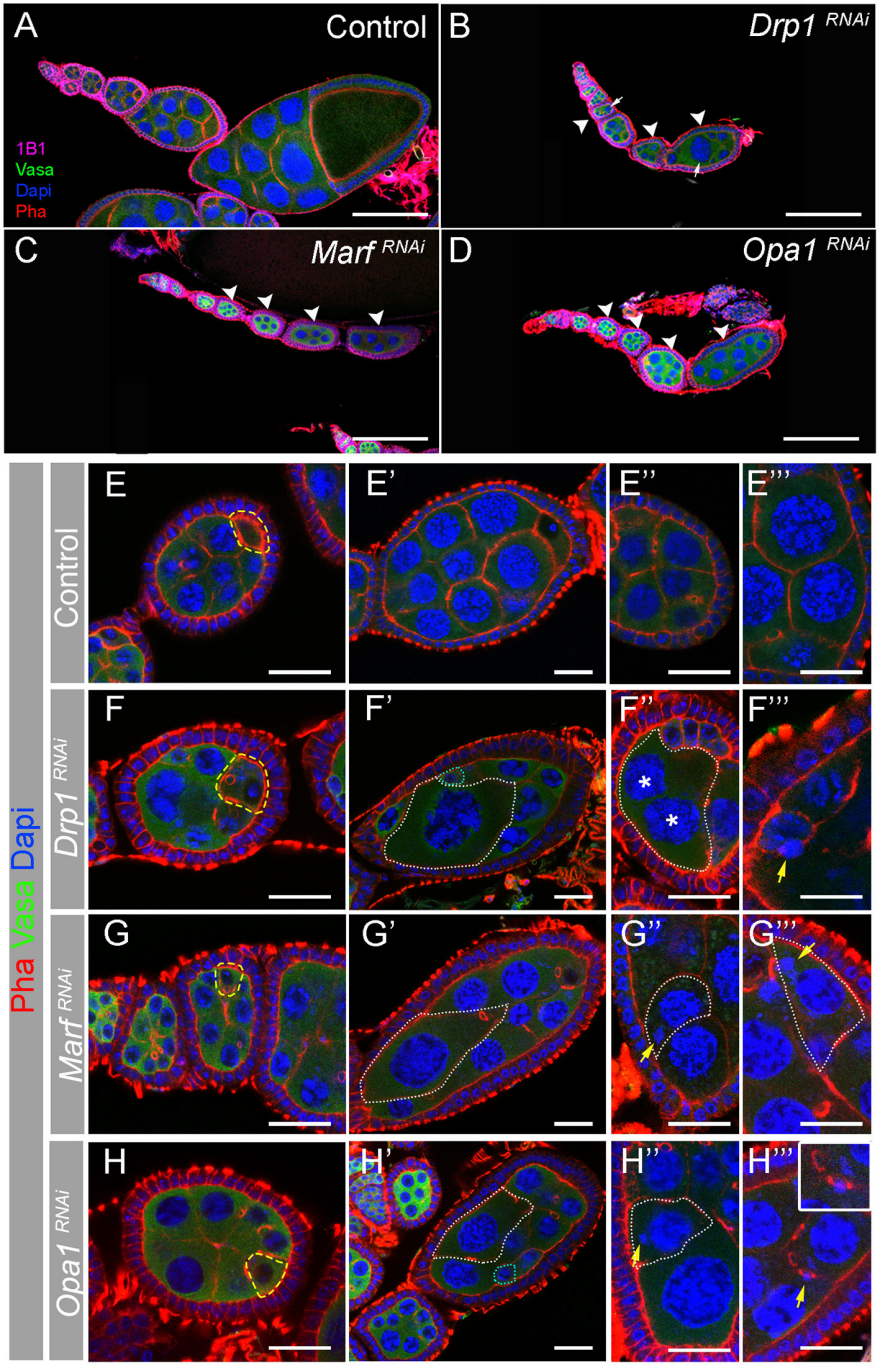
Depletion of *Drp1, Marf* , or *Opa1* induces multiple egg chamber defects. **(A–D)** Representative images of ovarioles from indicated RNAi driven by *nos*Gal4. Germ cells are marked with Vasa antibody (green). Fusomes are labeled with Hts (1B1, pink). Nuclei and F-actin are labeled by DAPI (blue) and Phalloidin (Pha, red), respectively. Scale bars represent 100 μm. Control (*mCherry*^*RNAi*^) ovariole **(A)** with a germarium in its anterior tip and a series of egg chambers progressively older. *Drp1*^*RNAi*^ **(B)**, *Marf*^*RNAi*^ **(C)**, or *Opa1*^*RNAi*^ **(D)** egg chambers degenerate before entering vitellogenic stages 8 and 9. When disrupting mitochondrial dynamics, egg chambers lose their normal structural organization presenting multiple defects in nurse cells morphology (arrowheads). **(E–H)** Several egg chamber defects are observed when mitochondrial dynamics regulators are knocked down. Abnormal egg chambers **(F–H)** are typically detected from egg chamber stage 3 or 4. Oocytes seem to be correctly specified based on the accumulation of F-actin in the posterior tip of egg chambers (outlined by dashed yellow line). **(E′-H′)** Egg chambers are severely disorganized in *Drp1*^*RNAi*^, *Marf*^*RNAi*^, and *Opa1*^*RNAi*^. Nurse cells show uneven cytoplasmic and nuclear size (white dashed lines). **(E″–H″, E**^**‴**^**–H**^**‴**^**)** Magnified areas show the variety of defects observed in abnormal egg chambers: multinucleated cells (**F″**, cell membrane is outlined by white line, and nuclei are labeled by asterisks) and DNA mislocalized in the cytoplasm **(G″,G**^**‴**^**,H″)**, in contact with cell membranes **(F**^**‴**^**)** or with ring canals (**H**^**‴**^, see magnified area). Scale bars represent 20 μm.

GSC polarized division is responsible for their self-renewal and for the formation of a daughter cell that is further away from the stem cell niche, the cystoblast that will divide several times. Hence, progressively more differentiated cells are located further away from GSCs. This results in an ovariole with undifferentiated cells at the most anterior tip, in the germarium, and more differentiated egg chambers at the most posterior side culminating with a fully developed egg (Figure 1). By individually knocking down *Drp1, Marf* , or *Opa1* in germ cells, we found that these ovarioles are shorter than control (Figures 4A–D), with later/more differentiated egg chambers being absent in most cases. We found that egg chamber development arrests by vitellogenic stage 8/9 and very rarely egg chambers progress to form a mature egg. Additionally, the egg chambers that are formed in these abnormal ovarioles have obvious morphological defects (Figures 4A–H).

A closer analysis of egg chambers in ovarioles where either RNAi targeting *Drp1, Marf* , or *Opa1* is expressed in the germline revealed that defects in egg chamber morphology are visible from very early on, with nurse cell sizes within the same egg chamber being abnormally variable (Figures 4E–H). Interestingly, in *Drp1*^*RNAi*^, *Marf*^*RNAi*^, and *Opa1*^*RNAi*^ egg chambers, the oocyte can be identified by the typical accumulation of F-actin (Figures 4E–H, yellow dashed line), indicating that the oocyte is specified and correctly positioned at the posterior side of stage 3/4 egg chambers. Surprisingly, as egg chamber development progresses, the asymmetries in nurse cell sizes become more obvious. Very large cells with enlarged nuclei can be observed, usually accompanied by the presence of abnormally small nurse cells with small nuclei (Figures 4E′–H′, dotted lines). The presence of nurse cells with large nuclei was very surprising and could potentially be a result of nuclei fusion events. However, in egg chambers with large cells/nuclei, we can identify a total of 16 nuclei (15 nurse cells and 1 oocyte, data not shown), therefore discarding the hypothesis of nuclear fusion. In some cases, multinucleated nurse cells can be observed (Figures 4E″,F″), indicating defects in membrane stability. In addition, a variety of DNA structure-related defects are also visible (Figures 4E″–H^‴^), with some nuclei appearing to be broken with small DNA fragments (DAPI positive) being separated from the nucleus (Figures 4G″,H″,F^‴^,G^‴^). In several cases, these smaller fragments are quite distant from the nucleus being observed close to cell membranes (Figure 4F^‴^) or in ring canals (Figure 4H^‴^). These findings suggest a functional link between mitochondrial dynamics and nurse cell morphology and may indicate a novel role of mitochondrial dynamics in nurse cell structure regulation.

**Figure 5:**
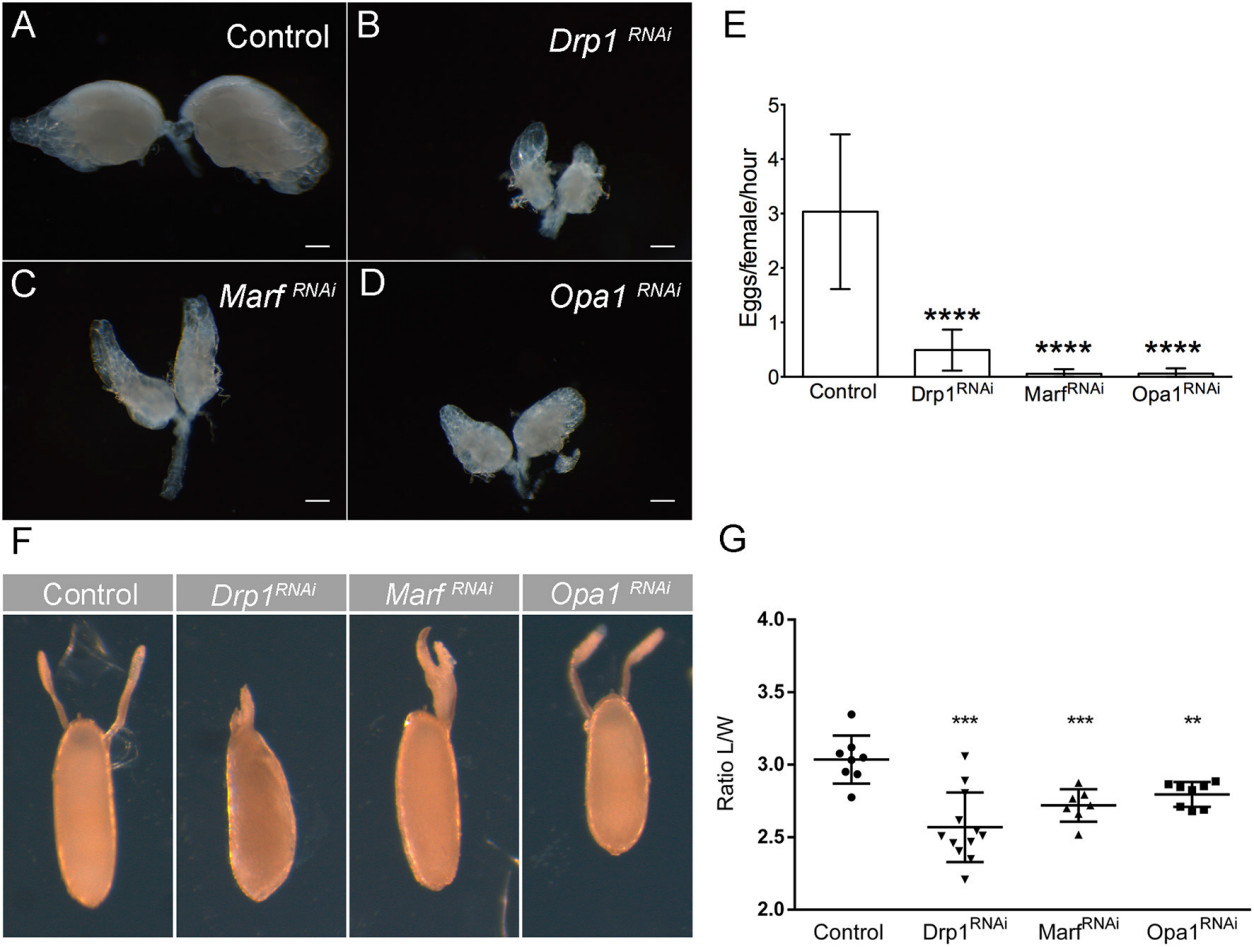
Knockdown of *Drp1, Marf* , or *Opa1* in GSCs leads to reduced fecundity. **(A–D)** Ovaries of 1-day-old female virgins expressing *Drp1*^*RNAi*^, *Marf*^*RNAi*^, or *Opa1*^*RNAi*^ in the germline by *nos*Gal4. **(A)** Bright-field images of whole ovaries of control (*mCherry*^*RNAi*^) females show normal ovary development and formation of mature eggs. **(B–D)** *Drp1*^*RNAi*^, *Marf*^*RNAi*^, or *Opa1*^*RNAi*^ females have tiny ovaries containing mainly germaria-like structures and generally lacking mature eggs. Scale bars represent 200 μm. **(E)** Quantification of the average number of eggs laid (±SD), per hour post-mating per female of the indicated genotype when crossed to wild-type males. Quantification revealed a significant decrease in fecundity of *Drp1*-, *Marf*-, and *Opa1*-knockdown females when compared with control (*mCherry*^*RNAi*^). **(F)** Representative images of dorsal appendage defects in eggs produced by females of the indicated genotypes. **(G)** Ratio Length/Width (L/W) (average ± SD) of the produced eggs. Eggs laid by *Drp1-, Marf-*, and *Opa1*-knockdown females show smaller L/W ratio indicating abnormal egg morphology. Statistical significance of differences compared with control was calculated using simple sample *t*-test. **P < 0.01; ***P < 0.001; ****P < 0.0001.

So far, we have established that mitochondrial dynamics is critical for the maintenance of GSC pool and correct egg chamber development in ovarioles. Next, we explored the physiological impact of mitochondrial dynamics on reproduction. Each female has two ovaries, composed by multiple ovarioles where the mature eggs are formed (Figure 1A). To determine how the defects in GSC number and egg chamber development, caused by deficient mitochondrial dynamics, affect female fertility, we started by analyzing ovarian morphology. Individual depletion of *Drp1, Marf* , or *Opa1* causes a dramatic reduction in ovary size (Figures 5A–D) with more differentiated egg chambers being mostly absent. We therefore asked whether these defects in ovaries could impact female fecundity. Indeed, the decrease in ovary size is consistent with a dramatic reduction in the number of eggs laid per female (Figure 5E). Interestingly, a closer analysis of the few eggs laid by females where *Drp1, Marf* , or *Opa1* is knocked down in germ cells revealed that these eggs have defective dorsal appendages with dorsal appendages' fusion being observed. Whereas two dorsolateral appendages are observed in control eggs, a single broad appendage is observed in *Drp1*^*RNAi*^ and *Marf*^*RNAi*^ (Figure 5F). Compared with control, eggs expressing *Drp1, Marf* , or *Opa1* RNAi have also smaller length to width ratio (Figure 5G), which translates into rounder eggs. These defects in the eggshell are an indication of defective egg chamber and oocyte development (Osterfield et al., 2017), which is consistent with the multiple defects observed in *Drp1*-, *Marf*-, or *Opa1*-depleted ovarioles (Figure 4).

These results show that mitochondrial dynamics in the female germline is not only essential for GSC regulation but is also essential during germline differentiation being required for the correct development of egg chambers, structural maintenance of nurse cells, egg formation, and ultimately female fertility.

### Downregulation of TCA Cycle Enzymes in GSCs Mimics the Phenotype Caused by Disruption of Mitochondrial Dynamics

Having identified mitochondrial dynamics as an important process in several stages of ovarian germ cell lineage development, we sought to further explore by which mechanism mitochondrial morphology could be playing a role.

**Figure 6:**
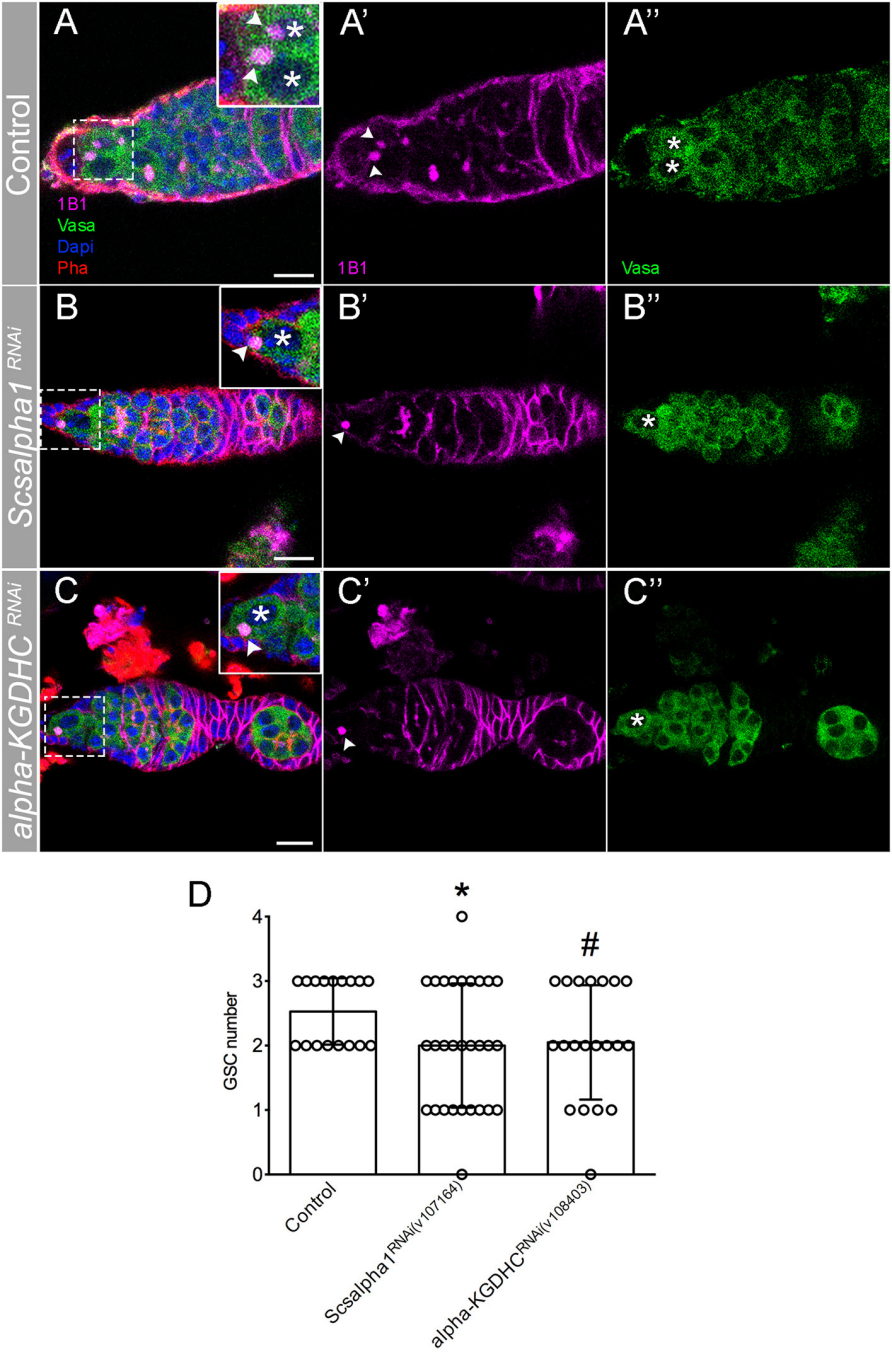
RNAi of TCA cycle enzymes leads to GSC loss. **(A–C)** Representative images of adult *Drosophila* germaria expressing *mCherry*^*RNAi*^ (control) **(A–A″)**, *Scsalpha1*^*RNAi*^ **(B–B″)**, or *alpha-KGDHC*^*RNAi*^ **(C–C″)** in the germline (*nos*Gal4). *Scsalpha1*^*RNAi*^ leads to a significant loss of GSCs. Despite not statistically significantly, *alpha-KGDHC*^*RNAi*^ also affects GSCs number. Spherical spectrosome (1B1, pink) identifies GSCs. Germ cells are labeled by Vasa antibody (green), nuclei by DAPI (blue), and F-actin by Phalloidin (Pha, red). Scale bars represent 10 μm. **(D)** Quantification of the average number of GSCs per germarium (±SD). Statistical significance of differences compared with control was calculated using simple sample *t*-test. *P < 0.05; ^#^*P* < 0.10.

It is known that during stem cell differentiation, OxPhos metabolism is favored over glycolysis (Rafalski et al., 2012). Since it has been shown that mitochondria morphology may be connected to energy metabolism in stem cells (Fang et al., 2016; Seo et al., 2020), we hypothesized that the changes in mitochondrial dynamics that occurs along germ cell lineage differentiation in the germarium could be necessary for the balance between glycolysis and OxPhos and therefore cell fate. In order to test this hypothesis, we depleted Succinyl-coenzyme A synthetase α subunit 1 [referred to as *scsalpha1*; UAS-*scsalpha1*^*RNAi*^ (v107164); knockdown ~90% validated by qPCR, this study] and E2 member of alpha-Ketoglutarate Dehydrogenase complex (referred to as *alpha-KGDHC*; UAS-*alpha-KGDH*^*RNAi*^ (v108403) as in Homem et al., 2014) in germ cells (*nos*Gal4). These are key regulatory enzymes of the TCA cycle whose activity levels were shown to be directly correlated with OxPhos (Tretter and Adam-Vizi, 2000; Phillips et al., 2009; Homem et al., 2014). Interestingly, RNAi-mediated depletion of both enzymes leads to a decrease in the GSC pool (Figures 6A–D), mimicking the phenotype caused by *Drp1*^*RNAi*^, *Marf*^*RNAi*^, and *Opa1*^*RNAi*^ (see Supplementary Table 2 for detailed characterization).

**Figure 7:**
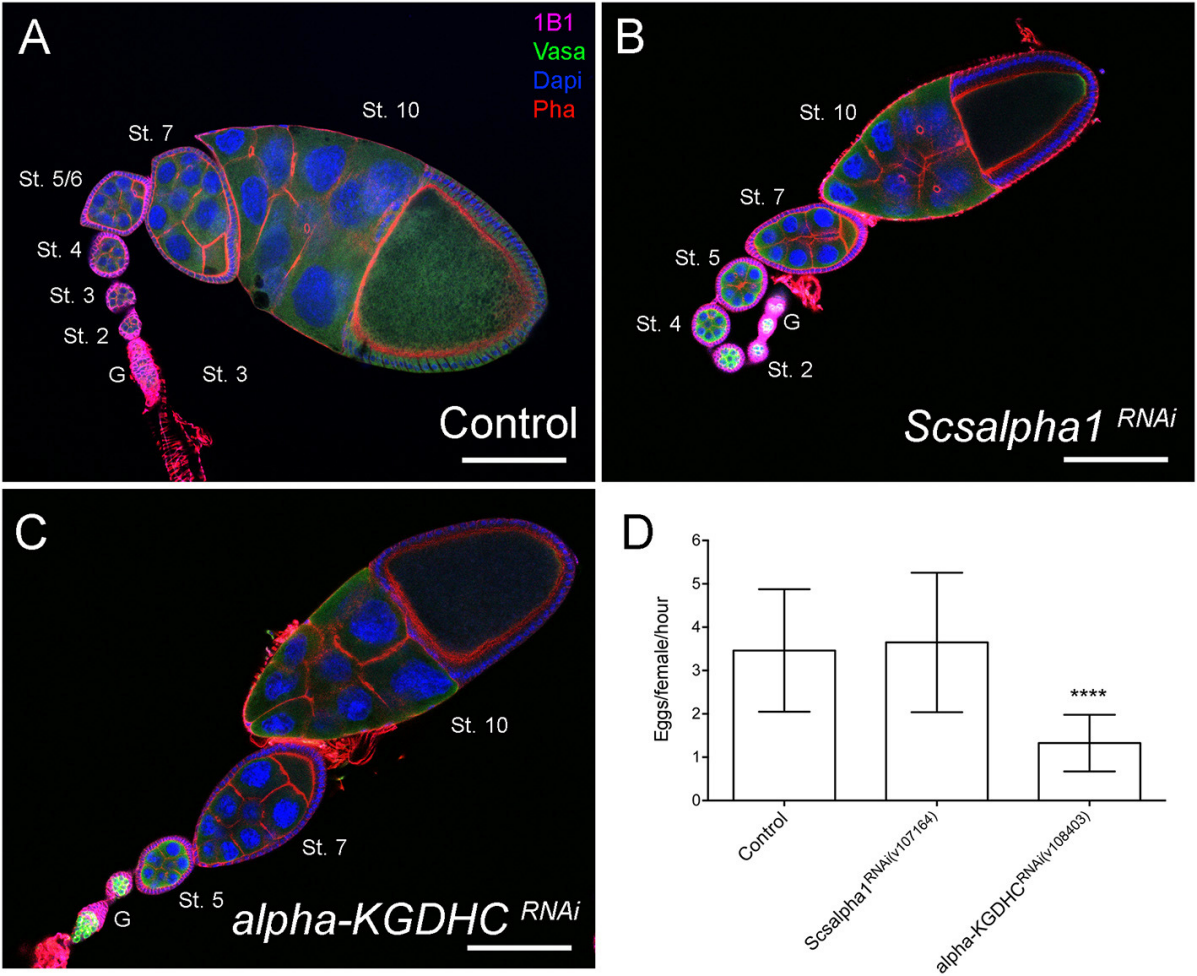
Downregulation of essential TCA cycle enzymes in the germline does not affect egg chamber morphology. **(A–C)** Representative images of ovarioles of indicated genotypes. Ovarioles of *Scsalpha1*^*RNAi*^ **(B)** or *alpha-KGDHC*^*RNAi*^ **(C)** show no obvious defects in egg chamber morphology compared with control (*mCherry*^*RNAi*^) **(A)**. Ovarioles of *alpha-KGDHC*^*RNAi*^ **(C)** present a reduced number of developing egg chambers comparing with control **(A)** or *scsalpha1*^*RNAi*^ **(B)**. Developmental stages of egg chambers are indicated (St.). Scale bars represent 100 μm. **(D)** Quantification of the average number of eggs laid (±SD) per hour post-mating, per female of the indicated genotype when crossed to wild-type males. Consistently, females expressing *alpha-KGDHC*^*RNAi*^ show decreased fertility. Statistical significance of differences compared with control was calculated using simple sample *t*-test. ****P < 0.0001.

However, contrary to knockdown of mitochondrial dynamics regulators, *scsalpha1*- and *alpha-KGDHC*-depleted ovarioles have normal egg chamber morphology with correct nurse cell organization and oocyte formation. These results indicate that mitochondrial dynamics may have an OxPhos-dependent role in GSCs and additional OxPhos-independent roles in nurse cells. Interestingly, while in ovarioles of control and *scsalpha1*^*RNAi*^ an average of eight distinct developmental stages are visible (Figures 7A,B), ovarioles of *alpha-KGDHC*^*RNAi*^ consistently present fewer chambers (only 3 or 4 stages), thus being considerably shorter (Figure 7C). This phenotype indicates delayed formation of novel egg chambers, possibly due to slower GSC division timings or lower survival of differentiated cystoblasts in *alpha-KGDHC*^*RNAi*^. Consistently, expression of *alpha-KGDHC*^*RNAi*^ in GSCs causes a significant reduction in the number of laid eggs, reducing female fecundity (Figure 7D). Interestingly, knockdown of *scsalpha1* in the germline is not sufficient to decrease female fecundity, suggesting that solely a reduction in GSC number without defects in ovariole development is not sufficient to compromise fecundity (Figure 7D). Both the ovaries and the eggs laid by *alpha-KGDHC*^*RNAi*^ or *scsalpha1*^*RNAi*^ females are morphologically indistinguishable from control (Supplementary Figures 2A,B), again confirming that interfering with TCA cycle alone is not sufficient to mimic the nurse cell defects caused by *Drp1*^*RNAi*^, *Marf*^*RNAi*^, and *Opa1*^*RNAi*^.

Together, these results surprisingly suggest that at least basal levels of TCA/OxPhos are required for the formation or maintenance of a stable GSC pool. Additionally, reducing TCA cycle enzyme levels in germ cells does not block germ cell differentiation in the germarium, although *alpha-KGDHC*^*RNAi*^ affects the pace of egg chamber formation. Contrary to depletion of fusion/fission regulators, knocking down TCA cycle enzymes does not lead to defects in nurse cell morphology, suggesting that the observed defects in older egg chambers are not only related to abnormal OxPhos levels.

## Discussion

**Figure 8:**
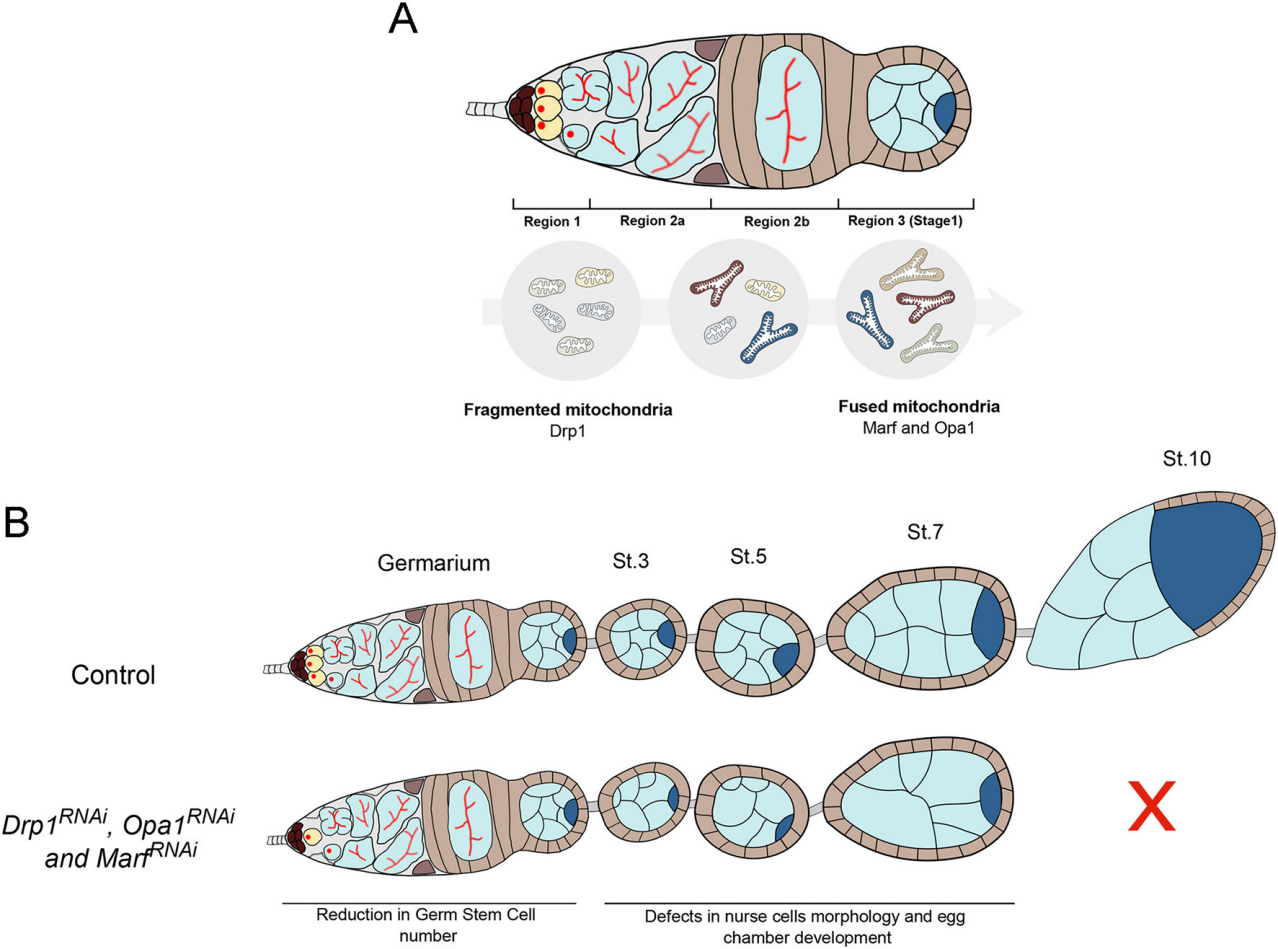
Mitochondrial dynamics is critical for *Drosophila* GSC maintenance and oogenesis. **(A)** GSCs in germarium region 1 have predominantly fragmented mitochondria. Throughout germ cell differentiation, mitochondria become more aggregated, indicative of increased fusion. These differential mitochondria morphologies along GSC lineage progression are regulated by fission regulator *Drp1* and fusion regulators *Marf* and *Opa1*. **(B)** Disruption of mitochondrial dynamics, by depletion of fission or fusion regulators in germ cells, causes defective mitochondria morphologies and GSC loss. Defective mitochondrial dynamics in the germline also causes severe defects in egg chambers that contain morphologically abnormal nurse cells and arrest their development before vitellogenic stages. Together, these defects in mitochondrial dynamics in the germline compromise egg chamber formation ultimately resulting in abnormal egg morphologies and reduced female fecundity.

Our work has revealed that both mitochondrial fusion and fission are required for the maintenance of the female GSC pool in *Drosophila* (Figure 8). Although GSCs predominantly have small, punctate mitochondria, we show that mitochondrial fusion is required in GSCs, indicating that the balance between fusion and fission also plays a functional role at this undifferentiated stage. The predominance of fissed mitochondria in GSCs and in region 1 of the germarium reported here is consistent with what has been observed in EM studies (Cox and Spradling, 2003). However, since stem cells are reported to mainly depend on glycolysis, to have low mitochondrial content and predominantly fissed mitochondria, our finding that mitochondria fusion events are an essential requirement in female GSCs is unexpected.

A closer analysis of mitochondrial activity revealed that GSCs normally have low mitochondrial membrane potential that increases with germline differentiation, consistently to what has been previously described (Wang et al., 2019). Interestingly, in a fraction of germaria depleted for either fusion or fission regulators, we observe the appearance of mitochondria with high levels of mitochondrial inner membrane potential that are never observed in the control situation. Notably, the phenotypes caused by *Opa1*^*RNAi*^, the regulator of inner mitochondrial fusion, are stronger than those originated by *Marf*^*RNA*^^i^, the regulator of outer-membrane fusion. Since *Opa1* has additional roles, other than fusion, being also important for mitochondria cristae shape and for maintaining ETC supercomplexes in the mitochondria cristae, this may explain the different outcomes (Cogliati et al., 2013). These results confirm that GSCs have functional mitochondria since a change in mitochondrial dynamics is capable of increasing mitochondrial activity. An increase in mitochondrial membrane potential could favor the formation of ROS; however, we did not find obvious evidence that GSC loss phenotypes are mediated by ROS. At first sight, these results might seem contradictory, but these bring to the spotlight the fact that, so far, no straightforward connection has been established between mitochondrial dynamics and bioenergetics, reported to depend on the cell type and context (reviewed in Liesa and Shirihai, 2013). Additionally, these results also support the notion that a fine balance between mitochondria fusion and fission is required to maintain a stable mitochondrial inner membrane potential and ensure healthy cell functions (Vazquez-Martin et al., 2012; Khacho et al., 2016; Luchsinger et al., 2016). Therefore, our data can contribute to the understanding of how the normal balance of mitochondrial dynamics is important for fate regulation and how its unbalance affects both mitochondria morphology, activity, and ultimately GSCs.

Even though the mechanism is unclear, mitochondria morphology is known to strongly impact the cell metabolic state, and several studies report fused mitochondria being associated with OxPhos metabolism (Rafalski et al., 2012; Mishra and Chan, 2016). The critical importance of fine tuning the levels of OxPhos and glycolysis for the regulation of stem cell fate could explain why mitochondrial dynamics may be fundamental in GSCs. Consistent with this hypothesis, we found that depletion of essential enzymes in the TCA cycle (*Scsalpha1* or *alpha-KGDHC*) also causes GSC loss, hence mimicking the defects caused by disrupting mitochondrial dynamics. Previous studies reported that GSCs express at low levels several members of the ETC (Kai et al., 2005) and have minor levels of mitochondrial respiration (Wang et al., 2019). Nevertheless, our results show that albeit at a minimal level, a functional TCA cycle, and probably OxPhos, is required in GSCs. This is in line with the currently accepted view that although stem cells primarily depend on glycolysis, these cells also require basal levels of OxPhos metabolism (Ito and Suda, 2014; Folmes and Terzic, 2016; Tsogtbaatar et al., 2020). Consistently, it has also been reported that human pluripotent stem cells possess functional respiratory complexes and are capable of consuming O_2_ at maximum capacity (Zhang et al., 2011), and that mouse pluripotent cells (mPSCs) require a certain degree of OxPhos to establish the primordial GSC identity (Bothun and Woods, 2020). Alternatively, one could argue that TCA cycle enzymes might play an additional unknown functional role in mitochondria maturation. However, this is unlikely since defects in mitochondria maturation do not cause GSC loss (Teixeira et al., 2015). Furthermore, mitochondria fusion is also known to be crucial for mitochondria quality control (Chan, 2012), allowing mixing of mitochondrial content to dilute damages, so our findings could reflect an accumulation of damaged mitochondria due to reduced fusion events, culminating in GSCs loss. Importantly, our results are consistent with what was recently observed in the male germline (Demarco et al., 2019) where depletion of mitochondrial fusion in GSCs results in GSC loss, suggesting that the requirement for basal levels of mitochondrial fusion is a common feature of *Drosophila* GSCs. Another noteworthy study showed that mitochondria fission regulator *Drp1* is also involved in aging-dependent GSC loss with an increase in fragmented mitochondria being associated with aged female GSC (Amartuvshin et al., 2020). This suggests that mitochondrial fusion is required for maintenance of female GSCs also during aging.

The work described here also shows that besides being important for GSC maintenance, mitochondrial dynamics is essential at later stages during germline differentiation. We found that impairment of mitochondrial fission or fusion leads to abnormal egg chambers with multiple defects in nurse cell morphology, culminating in the arrest of egg chamber development around vitellogenic stage 8/9. These defects in egg chambers at later stages were surprising, and this novel role for mitochondrial dynamics in nurse cell regulation is worth further exploration. One hypothesis is that abnormal fusion/fission events in the germline lead to defects in cyst formation and to defective oocyte specification. In the female germline, all differentiating cells are connected by ring canals and therefore exist in a syncytium. Once the oocyte is selected among the 16 cyst cells, all remaining 15 cells become nurse cells and transfer their mitochondria through ring canals into the oocyte to support its development (Cox and Spradling, 2003). Interestingly, a closer analysis of the previously published EM images of mitochondria in region 3 at the dumping stage (Cox and Spradling, 2003) shows that mitochondria are elongated and therefore predominantly fused, while crossing ring canals into the oocyte. Thus, abnormal mitochondria morphology could compromise mitochondria transfer, leading to a poorly developed oocyte that cannot progress further in oogenesis. In particular, we reported abnormal egg chambers showing obvious defects in nurse cell morphology, including the presence of highly variable cell and nuclei sizes, multinucleated cells, and also several DNA-related defects. Mitochondria are well-known sources of ROS as well as important regulators of intracellular calcium (Ca^2+^). Since ROS and Ca^2+^ levels play an important role in the regulation of actin cytoskeleton dynamics (Xu and Chisholm, 2014; Prudent et al., 2016; Hunter et al., 2018), impairment of mitochondria could explain the observed defects. Moreover, it was recently shown that mitochondrial fission modulator *Drp1* regulates F-actin dynamics during wound closure in the *Drosophila* epithelia (Ponte et al., 2020). In future studies, it would be interesting to test whether F-actin modulators are dysregulated and whether their impairment would result in similar egg chamber defects. On the other hand or in parallel, mitochondrial defects could lead to abnormal ROS levels, since mitochondrial-ROS production is highly dependent on organelle morphology (Galloway et al., 2012). High levels of ROS can lead to cellular oxidative stress and consequently to damages in DNA, lipids, and proteins (Rowe et al., 2008). Thus, the DNA defects observed in developing egg chamber upon *Drp1, Marf* , and *Opa1* downregulation in germ cells could possibly be explained by ROS-induced DNA damage and impaired DNA damage response (Srinivas et al., 2019). However, this is unlikely as we did not observe increased ROS levels in GSCs knocked down for mitochondrial dynamics regulators.

Reducing mitochondrial dynamics in germ cells ultimately results in reduced fecundity with the few eggs that are formed presenting an abnormal morphology. The morphology of the eggs and dorsal appendages directly results from late egg chamber shape, with eggshell components being secreted by follicle cells, which tightly surround the oocyte, mimicking its shape. Follicle cells are also responsible for the formation of the dorsal appendages or respiratory filaments, located at the dorsal-anterior end of the eggshell (Osterfield et al., 2017). Therefore, incorrect egg morphology indicates that egg chambers and oocyte did not develop as normal. Consistently, mitochondria morphology was also found to be important for proper oocyte development in mice, highlighting the critical role of mitochondria in oogenesis (Udagawa et al., 2014; Liu et al., 2016; Carvalho et al., 2020). Strikingly, decreasing the levels of OxPhos/TCA cycle enzymes in germ cells does not cause the same defects in late egg chamber development, nor in nurse cell morphology. These results indicate that the morphological defects in nurse cells observed when interfering with mitochondrial dynamics are not solely dependent on OxPhos. However, RNAi-mediated depletion of *alpha-KGDHC*, an enzyme of the TCA cycle, in germ cells leads to smaller ovarioles with the average ovariole presenting fewer developmental stages (3/4 stages vs. ~8 stages in control) and reduced fecundity. This phenotype could be caused by slower cycling of GSCs or lower survival of differentiated cystoblasts, which would then lead to sporadic formation and development of novel egg chambers. In combination these results suggest that, in GSCs, at least one of the roles of mitochondria is to maintain basal levels of TCA/OxPhos. Notwithstanding, in later stages of germline development, mitochondrial dynamics seem to play additional roles. This is consistent with the far-reaching influence of mitochondrial membrane potential, which is required not only for OxPhos but also for calcium storage, lipogenesis, activation of ROS and hypoxia-inducible factor (HIF), biogenesis of iron–sulfur clusters (ISCs), and mitochondrial protein import, among others (Picard et al., 2016).

Overall, our work highlights the importance of mitochondrial dynamics in the *Drosophila* female germline and the major impact of compromised fusion/fission events in oogenesis and consequently on fly fertility. We show that GSC number is regulated by both mitochondrial dynamics and TCA/OxPhos metabolism, suggesting that these two processes function together in these undifferentiated cells. Our results also reveal an OxPhos-independent role for mitochondrial dynamics in the regulation of nurse cell and egg chamber morphology, suggesting that mitochondria fusion and fission events play a broader role in oogenesis.

## Data Availability Statement

The original contributions presented in the study are included in the article/Supplementary Material, further inquiries can be directed to the corresponding author.

## Author Contributions

MG designed and performed experiments, data analysis, design of data analysis, figure assembly, and wrote the manuscript. JB-S designed and performed experiments, data analysis, design of data analysis, and figure assembly. PG performed experiments, data analysis, and manuscript editing. CH did conceptual design of the study, designed experiments, data analysis, assisted figure assembly, and wrote the manuscript. All authors contributed to the article and approved the submitted version.

## Conflict of Interest

The authors declare that the research was conducted in the absence of any commercial or financial relationships that could be construed as a potential conflict of interest.
