# Supplementary material for: Mitochondrial Dynamics in the Drosophila Ovary Regulates Germ Stem Cell Number, Cell Fate, and Female Fertility
Source: Front Cell Dev Biol. 2021 Jan 28;8:596819. doi: 10.3389/fcell.2020.596819 (PMC7876242; doi:10.3389/fcell.2020.596819)
Supplement: Supplementary file 1 [file Data_Sheet_1.pdf]

Supplementary Material

1 Supplementary Tables

1.1 Supplementary Table 1

Table 1. List of *Drosophila* lines used in the study.

Function	Gene/cell type affected	Genotype	Source	Library
**Control**	**mCherry**	y[1] sc[*] v[1] sev[21]; P{y[+t7.7] v[+t1.8]=VALIUM20-mCherry}attP2	BL35758	TRiP, V20
**Driver**	**Germ cells**	P{w[+mC]=UAS-Dcr-2.D}1, w[1118]; P{w[+mC]=GAL4-nos.NGT}40	BL25751	-
**Mitochondrial dynamics**	***Drp1***	y1 v1; P{TRiP.HMC03230}attP40	BL51483	TRiP, V20
		w1118; P{UAS-Drp1.miRNA.CDS.S}3/T(2;3)TSTL, CyO: TM6B, Tb1	BL67160	miRNA
		w1118; P{GD10456}v44155/TM3	v44156	GD
	Marf	y1 sc* v1; P{TRiP.HMC03883}attP40	BL55189	TRiP, V20
		w1118; P{UAS-Marf.miRNA.CDS.S}3	BL67158	miRNA
		w1118; P{GD11094}v40478	v40478	GD
		w1118; P{KK105681}VIE-260B	v105261	KK
	Opa1	y1 sc* v1; P{TRiP.HMS00349}attP2	BL32358	TRiP, V20
		w1118; Bl1/CyO; P{UAS-Opa1.miRNA.CDS}3	BL67159	miRNA
		w1118; P{KK105706}VIE-260B	v106290	KK
**TCA cycle**	***Scsalpha1***	w1118; P{KK102542}VIE-260B	v107164	KK
	alpha-KGDHC	w1118; P{KK109081}VIE-260B/cyo;Dr/tm3ser	v108403	KK

V20, Valium 20

1.2 Supplementary Table 2

Table 2. Descriptive summary of downregulation effects of mitochondrial dynamics-regulator genes in GSC number, fate and fecundity of female *Drosophila*.

Gene	Fly line	GSCs in the germarium			Mitochondrial morphology in the germarium		Egg chamber formation	Ovary size	Egg morphology	Fecundity	
		Region 1									
		GSC number (mean± SD)	P-value vs Control	Germaria analyzed	Region 1	Regions 2b-3				Egg/female/h (mean±SD)	P-value vs Control
**Control**	BL35758	2.53±0.51	-	17	Mostly fissed	Mostly fused	normal	normal	normal	3.04±1.42	-
Drp1	v44156	1.46±0.99	0.0002	28	Mostly fused	Mostly fused	Smaller ovarioles; Defects in nurse cells	tiny	Smaller eggs; Defective dorsal appendages	0.49±0.38	< 0.0001
	BL51483	2.00±1.05	0.0603	28	n.d.	n.d.	n.d.	tiny	n.d.	n.d.	n.d.
	BL67160	1.65±0.65	< 0.0001	34	n.d.	n.d.	n.d.	tiny	n.d.	n.d.	n.d.
Marf	v40478	2.00±0.65	0.0074	25	Mostly fissed	Mostly fissed	Smaller ovarioles; Defects in nurse cells	tiny	Smaller eggs; Defective dorsal appendages	0.05±0.08	< 0.0001
	v105261	2.20±0.61	0.0894	20	n.d.	n.d.	n.d.	tiny	n.d.	n.d.	n.d.
	BL55189	2.81±0.40	0.0897	16	n.d.	n.d.	n.d.	tiny	n.d.	n.d.	n.d.
	BL67158	1.95±0.71	0.0084	19	n.d.	n.d.	n.d.	tiny	n.d.	n.d.	n.d.
Opa1	v106290	1.67±0.85	0.0004	33	Mostly fissed	Mostly fissed	Smaller ovarioles; Defects in nurse cells	tiny	Smaller eggs	0.06±0.10	< 0.0001
	BL32358	1.71±0.89	0.0009	38	n.d.	n.d.	n.d.	tiny	n.d.	n.d.	n.d.
	BL67159	2.23±0.81	0.1868	26	n.d.	n.d.	n.d.	tiny	n.d.	n.d.	n.d.
Scsalpha1	v107164	2.00±0.96	0.0424	29	n.d.	n.d.	normal	normal	normal	3.64±1.61	>0.10
alpha-KGDHC	v108403	2.05±0.89	0.0576	20	n.d.	n.d.	Smaller ovarioles	normal	normal	1.33 ±0.65	< 0.0001

n.d., not determined

1. Supplementary Figures

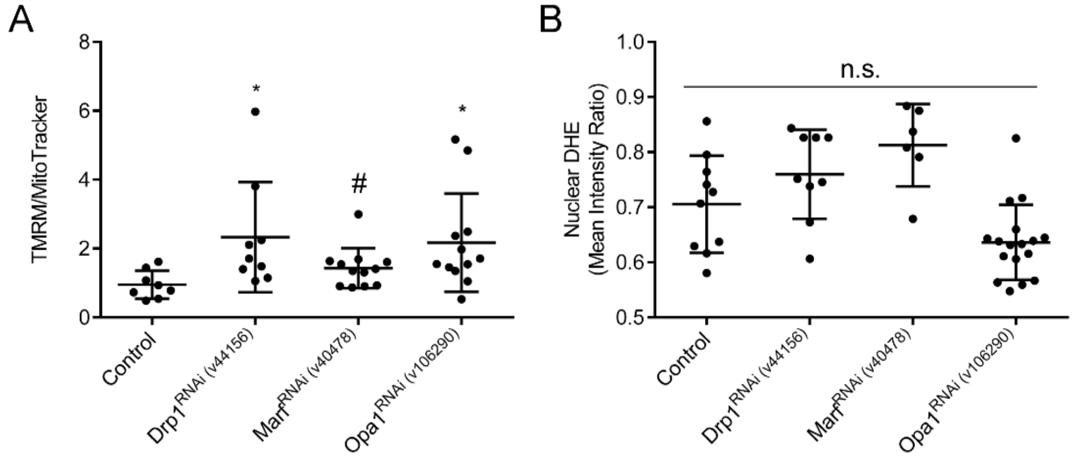

Supplementary Figure 1. Knock-down of *Drp1*, *Marf* or *Opa1* alters mitochondrial membrane potential but does not induce significant changes in ROS levels in GSCs. (A) Quantification of TMRM/MitoTracker in region 1 of the germaria of females of the indicated genotypes. TMRM/MitoTracker intensity ratios were calculated in region 1 of germaria expressing *Drp1*^{RNAi} $^{(v44156)}$, *Marf*$^{RNAi\ (v40478)}$ or *Opa1*$^{RNAi\ (v106290)}$ in GSCs, under the regulation of *nos*GAL4. Interfering with both fusion or fission regulators significantly increase the mitochondrial membrane potential in GSCs. **(B)** Dihydroethidium (DHE) staining was used to assess the level of ROS upon *Drp1*, *Marf* or *Opa1* depletion in GSCs. The mean intensity of nuclear DHE relative to cytoplasm was quantified in cells within region 1 of the germaria. The resulting data is represented in the graph. Disruption of mitochondrial dynamics (*Drp1*-, *Marf*- or *Opa1*- RNAi) did not lead to an obvious change in ROS when compared to control (*mCherry*^{RNAi}). The ratio values, mean and ±SD are depicted in the graphs. Statistical significance of differences to control was calculated using *Dunn*'s multiple comparisons test (DHE) or unpaired t-test (TMRM/MitoTracker). *P < 0.05, **P < 0.01, #P < 0.10, n.s. non-significant.

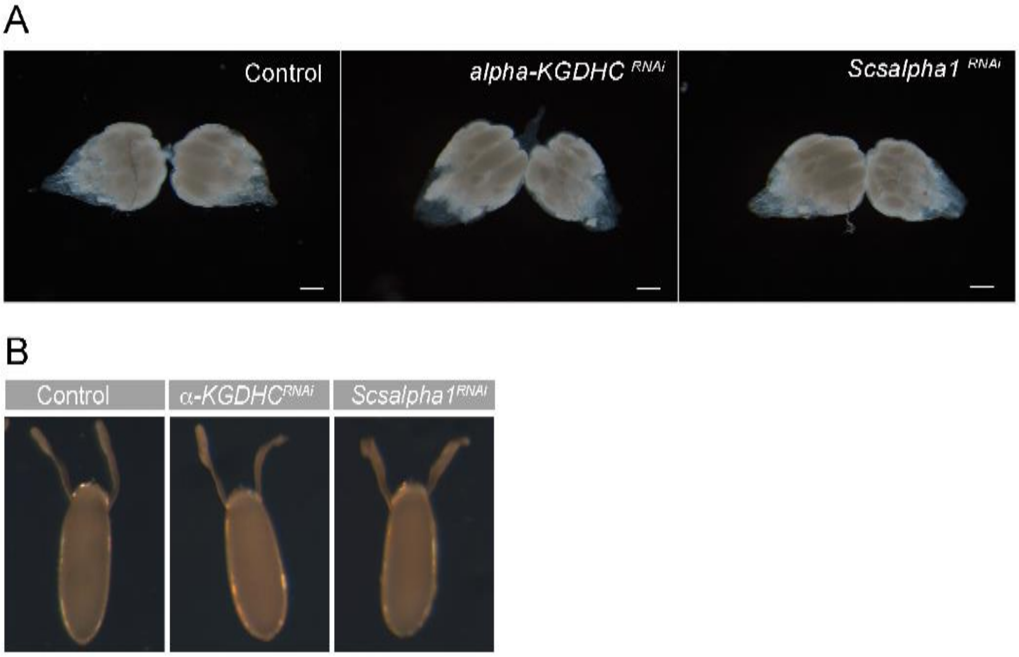

Supplementary Figure 2. Downregulation of essential TCA cycle enzymes in the germline does not affect ovaries or egg morphology (A) Bright-field images of whole ovaries of one-day-old female virgins of the indicated genotypes. Germline cells expressing either *Scsalpha*^{RNAi} or *alpha-KGDHC*^{RNAi} under the control of *nos*Gal4 generate wild-type like ovaries. Scale bars represent 200 μm. **(B)** Representative images of the eggs produced by females of the indicated genotypes. Eggs produced by *Scsalpha*^{RNAi} or *alpha-KGDHC*^{RNAi} females are morphologically similar to eggs laid by control flies (*mCherry*^{RNAi}).
